# Autophagy, a critical element in the aging male reproductive disorders and prostate cancer: a therapeutic point of view

**DOI:** 10.1186/s12958-023-01134-1

**Published:** 2023-09-26

**Authors:** Pourya Raee, Shing Cheng Tan, Sajad Najafi, Farshid Zandsalimi, Teck Yew Low, Shahin Aghamiri, Elham Fazeli, Mahyar Aghapour, Zahra Shams Mofarahe, Mohammad Hossein Heidari, Fatemeh Fadaei Fathabadi, Farid Abdi, Mohsen Asouri, Ali Asghar Ahmadi, Hossein Ghanbarian

**Affiliations:** 1 Student Research Committee, Department of Biology and Anatomical Sciences, School of Medicine, Shahid Beheshti University of Medical Sciences, Tehran, Iran; 2 UKM Medical Molecular Biology Institute, Universiti Kebangsaan Malaysia, Kuala Lumpur, Malaysia; 3 Department of Medical Biotechnology, School of Advanced Technologies in Medicine, Shahid Beheshti University of Medical Sciences, Tehran, 19395-4719 Iran; 4 Department of Molecular Medicine, School of Advanced Technologies in Medicine, Tehran University of Medical Sciences, Tehran, Iran; 5 Student Research Committee, Department of Medical Biotechnology, School of Advanced Technologies in Medicine, Shahid Beheshti University of Medical Sciences, Tehran, Iran; 6 Cellular and Molecular Biology Research Center, Shahid Beheshti University of Medical Sciences, Tehran, Iran; 7 Mehr Fertility Research Center, Guilan University of Medical Sciences, Rasht, Iran; 8 Department of Dermatology and Allergic Diseases, Ulm University, Ulm, Germany; 9 Department of Biology and Anatomical Sciences, School of Medicine, Shahid Beheshti University of Medical Sciences, Tehran, Iran; 10 Department of Chemical Engineering, Science and Research branch, Islamic Azad University, Tehran, Iran; 11 North Research Center, Pasteur Institute of Iran, Amol, Iran; 12 Urogenital Stem Cell Research Center, Shahid Beheshti University of Medical Sciences, Tehran, Iran

**Keywords:** Aging, Autophagy, Erectile dysfunction, Inflammaging, Male infertility, Prostate cancer

## Abstract

Autophagy is a highly conserved, lysosome-dependent biological mechanism involved in the degradation and recycling of cellular components. There is growing evidence that autophagy is related to male reproductive biology, particularly spermatogenic and endocrinologic processes closely associated with male sexual and reproductive health. In recent decades, problems such as decreasing sperm count, erectile dysfunction, and infertility have worsened. In addition, reproductive health is closely related to overall health and comorbidity in aging men. In this review, we will outline the role of autophagy as a new player in aging male reproductive dysfunction and prostate cancer. We first provide an overview of the mechanisms of autophagy and its role in regulating male reproductive cells. We then focus on the link between autophagy and aging-related diseases. This is followed by a discussion of therapeutic strategies targeting autophagy before we end with limitations of current studies and suggestions for future developments in the field.

## Introduction

Autophagy refers to an evolutionarily conserved cellular process in which redundant or dysfunctional cellular components are degraded and recycled by a lysosome-dependent mechanism [1]. The concept of autophagy is not new, as the term was first described by the French physiologist M. Anselmier in 1859 and was taken up a century later by Christian de Duve, the founding father of modern lysosome and autophagy research, at a symposium in 1963 [2]. Moreover, the regulatory process of autophagy and the associated autophagy-related genes (ATGs) have been precisely defined since 1993 by Yoshinori Oshumi using mutant strains of *Saccharomyces cerevisiae* [3]. The importance of autophagy in physiological processes and diseases is reflected in the fact that Yoshinori Oshumi was awarded the Nobel Prize in Physiology and Medicine in 2016 for discovering the mechanisms of autophagy.

Autophagy differs from the ubiquitin-proteasome system, which selectively degrades only ubiquitinated biomolecules [1]. The latter also degrades short-lived proteins, in contrast to the autophagy process, which is predominantly responsible for the degradation of long-lived proteins. Autophagy is mainly associated with starvation, whereby cells deplete their energy reserves to survive until nutrients are replenished. In this process, long-lived or dysfunctional cytoplasmic proteins are degraded in the lysosomes and recycled [4]. Autophagy occurs continuously, even under physiological conditions to protect cells from nutrient deficiency. Therefore, it is essential to know that whereas “baseline autophagy” occurs constitutively to regulate the turnover of cytoplasmic components, “induced autophagy” is triggered under certain conditions, such as starvation, to produce amino acids that help the organism adapt to the particular conditions [5].

Apart from the role of autophagy in cellular homeostasis, autophagy is critical in combating disease and developing functional biological structures, as well as promoting health and longevity. It is well known that aberrant regulation of autophagy can lead to diseases [6]. A new trend in this field is the impact of autophagy dysregulation on male reproductive health. Nowadays, men encounter many reproductive health problems, including reduced fertility potential, ejaculatory dysfunction, penile disorders, impotence, and urological cancers [7–9]. This is a worrying trend because men have a shorter life expectancy and are less likely to seek medical care [10, 11]. Studies in 1992 and 2017 found consistent declines in sperm counts in the global male population [12, 13].

Furthermore, several studies have linked male subfertility to somatic and general health status [14, 15]. For example, a Danish cohort study of 4,712 men found that semen quality can be used as a predictor of long-term morbidity [16]. This was supported by another systematic review that confirmed this association with concomitant health problems such as urogenital malignancies, testicular cancer, diabetes mellitus, cardiovascular disease, and other metabolic disorders [17]. In this review, we discuss the role of autophagy in aging male reproductive disorders, including prostate cancer (PCa).

## Mechanisms of autophagy

**Fig. 1:**
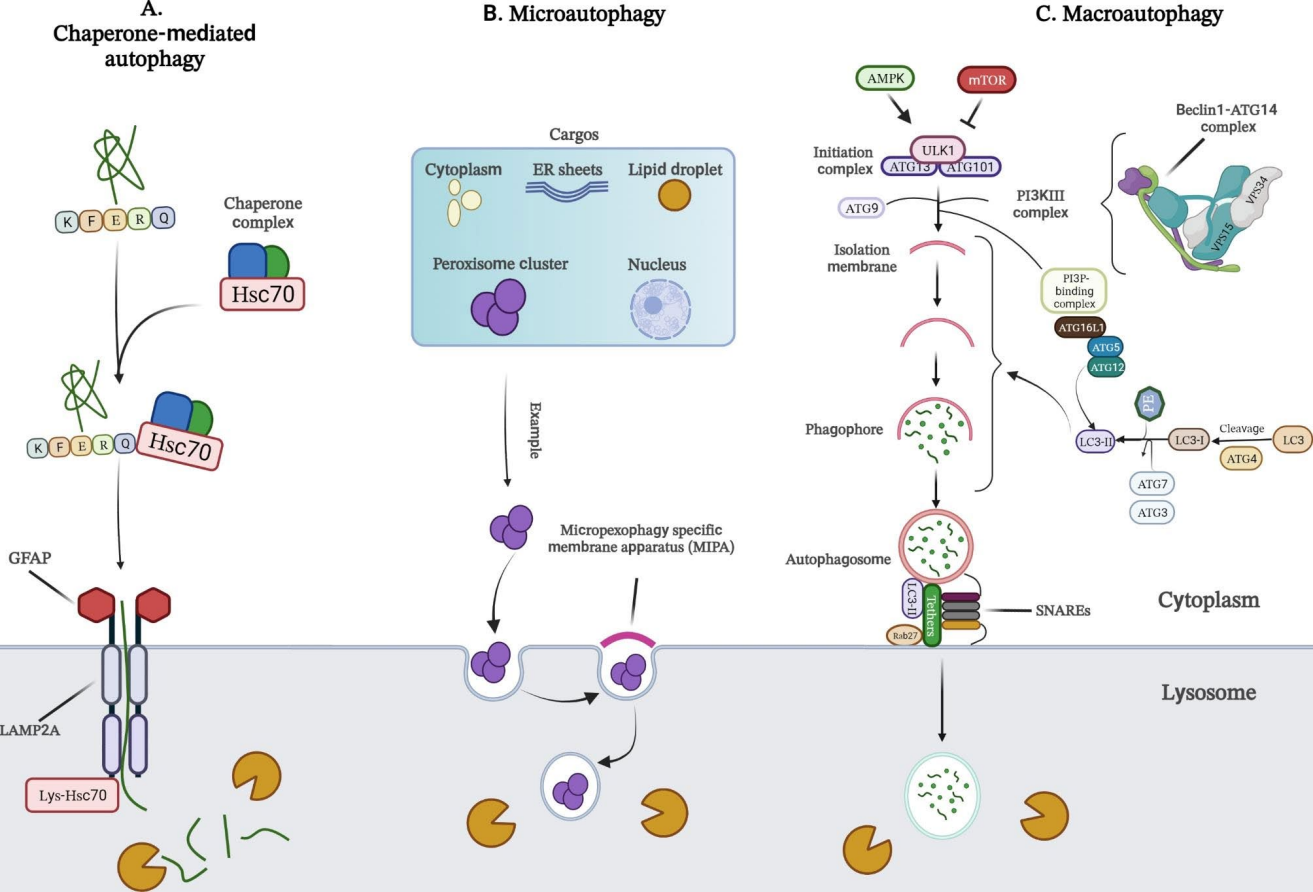
**A-C**) Schematic representation of three main types of autophagy process, including chaperone-mediated autophagy (**A**), microautophagy (**B**), and macroautophagy (**C**). HSC70: heat shock cognate 71 kDa protein; GFAP: glial fibrillary acidic protein; LAMP2A: lysosome-associated membrane protein type 2 A; ER: endoplasmic reticulum; lys-HSC70: lysosomal HSC70; AMPK: AMP-activated protein kinase; mTOR: mammalian target of rapamycin; ATG: autophagy-related proteins; ULK1: Unc-51-like kinase 1; PI3K: phosphoinositide 3-kinase; PI3P: phosphatidylinositol 3-phosphate; LC3: microtubule-associated protein 1 A/1B-light chain 3; PE: phosphatidylethanolamine

Depending on the mechanisms by which cargo is delivered to lysosomes, biologists have classified autophagy into three types, namely (i) macroautophagy, (ii) microautophagy, and (iii) chaperone-mediated autophagy (CMA) (Fig. 1). Macroautophagy is an autophagosome-dependent autophagy. Autophagosomes are cytosolic double-membrane vesicles. They sequester and transport cargo to lysosomes by engulfing a portion of the cytoplasm containing components to be degraded, such as cytosolic proteins, organelles, and microorganisms. In microautophagy, on the other hand, the lysosomal membrane forms an invagination to allow direct uptake of the cargo. Finally, in CMA, KFERQ-like motif-containing proteins are digested by lysosomes after identification by cytosolic chaperones. Ultimately, all three transport mechanisms end with cargo degradation and transfer of degradation products to the cytosol for recycling [18–22]. The best-studied type of autophagy is macroautophagy, which will be referred to simply as “autophagy” in the following paragraphs.

Key regulators of autophagy include mammalian target of rapamycin (mTOR) and AMP-activated kinase (AMPK), which inhibits and activates the process, respectively (Fig. 1C). When autophagy is triggered, phagophores (a cup-shaped structure) are first formed, followed by sequestration of the autophagic cargo into autophagosomes, which consist of double-membrane vesicles. Subsequently, these autophagosomes fuse with acidic lysosomes to form autolysosomes, where degradation of the autophagic cargo occurs. The autophagy process can be summarized in five steps: (i) initiation, (ii) nucleation of the membrane and formation of phagophores, (iii) expansion of phagophores, (iv) fusion with the lysosome, and (v) degradation. Each step is controlled by a group of proteins, the ATGs, which can form several protein complexes [20]. Some examples of these protein complexes are the Unc-51-like kinase 1 (ULK1, also known as Atg1) initiation complex, the class III phosphatidylinositol 3-kinase (PI3K) nucleation complex, and the phosphatidylinositol 3-phosphate (PI3P) binding complex. These complexes facilitate autophagosome formation by regulating the transfer of various proteins, such as ATG12 and light chain 3 (LC3, also known as ATG8 in yeast). ATG12 then binds to ATG5, which in turn binds to ATG16L1. This complex then dimerizes and interacts with the PI3P-binding complex to promote the conjugation of LC3. Subsequently, LC3 is cleaved by the protease ATG4 to form LC3-I, which is then conjugated with phosphatidylethanolamine (PE) to form LC3- II, which is incorporated into pre-autophagosomal and autophagosomal membranes. These membranes are formed, at least in part, by ATG9-containing vesicles. The final step in the autophagy process is the fusion of the autophagosome and lysosome, followed by the degradation of the cargo [20, 23].

In addition to nutrient deficiency or starvation, other external and intracellular stimuli such as hypoxia, reactive oxygen species (ROS), aggregation of dysfunctional proteins, and damaged organelles are known to stimulate autophagy [24]. As such, autophagy can be regulated by two main systems: (i) the nitrogen-dependent system, which relies on the TOR signaling pathway, and (ii) the energy-dependent system, which depends on the AMPK pathway that acts as a biological sensor of the availability of nitrogen and glucose concentration [25].

## Autophagy and the regulation of male reproductive cells

### Spermatozoa

Mammalian germ cell development is highly regulated in the seminiferous tubules of the male testis. Spermatogenesis can be divided into three discrete phases: (i) mitotic proliferation, in which spermatocytes develop from undifferentiated spermatogonia; (ii) meiotic division, in which spermatocytes undergo cell divisions to generate haploid spermatids; and (iii) the spermiogenesis phase, in which haploid round spermatids undergo a complex process of morphological changes and structural reorganization to form mature spermatozoa [26]. These include condensation and remodeling of nuclear chromatin, acrosome biogenesis, formation of the mitochondrial envelope, removal of excess cytoplasm, remodeling of the centriole, and construction of sperm flagella to form elongated spermatids and spermatozoa. The final step is a process known as spermiation, in which mature spermatozoa are released into the lumen of the seminiferous tubules before entering the epididymis [27, 28]. Disruption or alteration of these processes results in abnormal spermatid differentiation and can lead to defects in sperm morphology [29].

Autophagy is involved in regulating the physiological and pathophysiological conditions at each phase of spermatogenesis [26]. This is reflected in the fact that proteins related to the autophagy pathway, such as ATG5, Atg16, LC3, AMPKα1/2, m-TOR, Beclin 1, PINK1, and p62, are active in human spermatozoa [30]. At the physiological level, autophagy plays a cytoprotective role at different stages of spermatogenesis, from spermatogonia to mature spermatozoa [31–38]. Activation of autophagy also increases sperm motility [30]. On the other hand, it has been shown that under certain stress conditions, such as the mouse model of heat stress, accelerated autophagy triggers an apoptotic cascade in germ cells, leading to decreased germ cell viability [39].

**Fig. 2:**
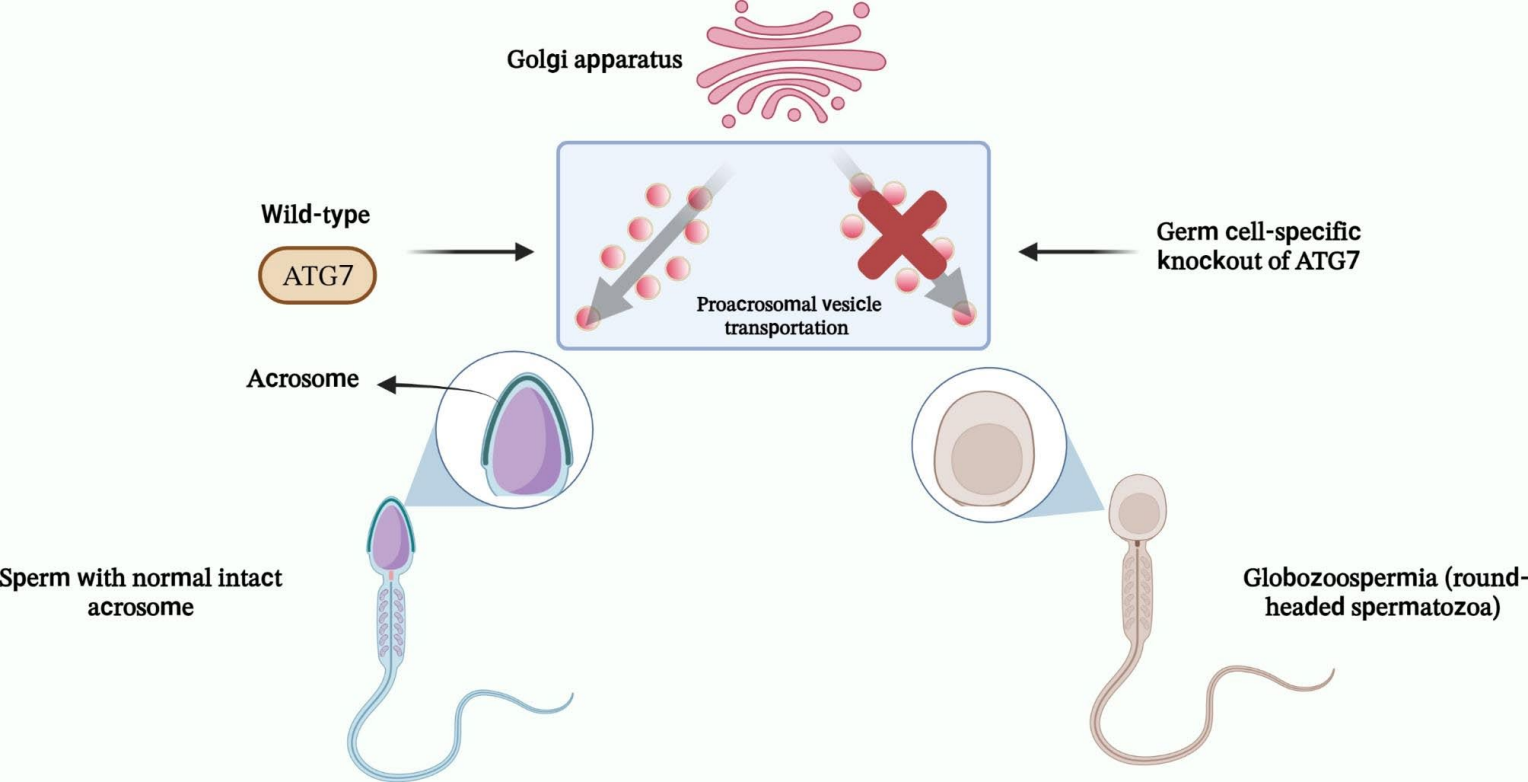
Proposed role of ATG7 in acrosome biogenesis. ATG7 is involved in acrosome biogenesis by regulating the transport of proacrosomal vesicles and/or fusion with the acrosome. Germ cell-specific knockout of this gene in mouse models could result in the failure of acrosome biogenesis, which corresponds to globozoospermia in humans

Autophagy is also involved in the biogenesis of the acrosome (Fig. 2), a Golgi-derived secretory organelle with a cap-like structure located over the anterior part of the sperm head and containing several proteolytic and hydrolytic enzymes similar to the lysosome [28]. The release of the acrosomal enzyme, a process known as acrosomal reaction, is an essential step for fertilization as it allows sperm to pass through the zona pellucida (ZP) and eventually interact with the oocyte’s plasma membrane [40]. It has been reported that male mice with germ cell-specific knockout of ATG7 exhibit almost complete infertility due to failure of acrosome biogenesis, which corresponds to globozoospermia in humans (Fig. 2). ATG7 has been proposed to be involved in acrosome biogenesis by regulating the transport of proacrosomal vesicles and/or fusion with the acrosome [41]. In addition to acrosome biogenesis, researchers have demonstrated the involvement of autophagy in regulating cytoplasmic degradation and spermatozoan flagella biogenesis during spermiogenesis. In ATG7-null spermatozoa, there are alterations in the ‘9 + 2’ structure of sperm flagella and insufficient removal of cytoplasm caused by abnormal accumulation of PDZ and LIM domain 1 (PDLIM1), a negative regulator of cytoskeletal organization. Thus, disruption of F-actin and microtubules leads to a manchette structure defect, insufficient cytoplasm removal, and abnormal tail axis assembly [42]. Another study has revealed that the disruption of ATG5 in mouse germ cells resulted in decreased autophagic flux. This condition led to multiple spermiogenesis abnormalities, including compromised acrosome biogenesis, disrupted mitochondrial rearrangement, and the accumulation of excessive cytoplasm and residual bodies [43]. Furthermore, it was shown that transcriptional factor EB (TFEB), a major regulator of lysosomal biogenesis, autophagy, and endocytosis, is differentially expressed and activated across different regions of mouse testes, which may facilitate cell migration across the blood-testis barrier (BTB) [44]. According to what we have discussed so far, autophagy plays an essential role in spermatogenesis.

### Sertoli cells

The sperm epithelium contains the Sertoli cells (SCs), which can control spermatogenesis. Germ cell development requires an intact environment as well as structural support from the BTB, which depends on the ectoplasmic specialization (ES) of SCs [26, 28, 45]. ES, an anchoring junction rich in actin microfilament, is divided into two distinct compartments, the apical ES and the basal ES. The main function of the apical ES is to shape the spermatid head, ensure proper spermatid movement, orient the elongated spermatids, and regulate the process of spermiation. The basal compartment is the constructive part of the BTB [26, 28, 45].

**Fig. 3:**
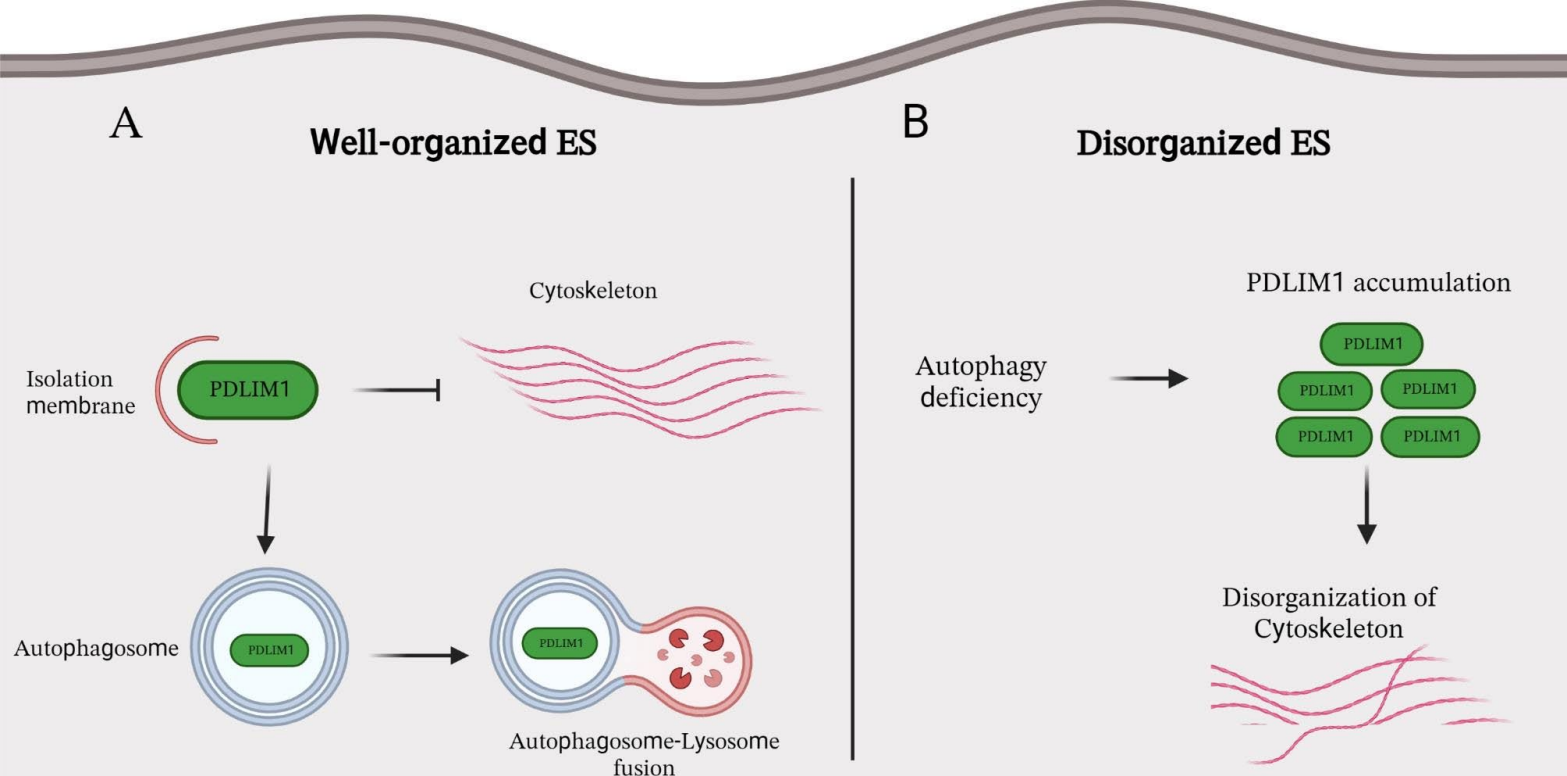
The involvement of autophagy in the organization of ES in Sertoli cells. The apical and basal ES are actin microfilament-rich anchoring junctions. The apical ES shapes the spermatid head, controls spermiation, and orients elongated spermatids. The basal compartment is the constructive part of the BTB. ES: ectoplasmic specialization; PDLIM1: PDZ and LIM domain 1; BTB: blood-testis barrier

Silencing of genes related to autophagy has been shown to lead to the degradation of apical and basal ES and the disorganization of cytoskeletal structures in mouse models, resulting in the production of a greater number of spermatozoa with deformed heads. Current evidence suggests that PDLIM1 is the primary target of autophagy-mediated degradation during the ES assembly process (Fig. 3). Therefore, a lack of autophagy results in abnormal ES structure due to the accumulation of PDLIM1 in SCs [46]. Moreover, several studies have shown that upregulated autophagy as an anti-apoptotic/anti-necrotic signaling pathway contributes to the survival of SCs and germ cells under stress conditions [47–50]. It was reported that ULK1, a protein kinase involved in autophagy activation, is essential for regulating goat SCs viability [51]. Autophagy also regulates the elimination of testis-specific sex hormone-binding globulin (SHBG), also known as androgen-binding protein (ABP), in SCs under the control of testosterone. Testosterone increases the expression of ABP by inhibiting autophagic degradation [52]. On the other hand, activation of autophagy has been shown to be necessary for the clearance of apoptotic germ cells by SCs [53].

The discovery of the participation of microRNAs (miRNAs) in the scenario raised interest in our knowledge of the functions of autophagy in SCs. For instance, the elimination of apoptotic germ cells through LC3-associated phagocytosis was shown to be regulated by interactions between miR-471-5p and autophagy-related proteins. Elevated germ cell apoptosis has been reported in mouse models engineered to express this miRNA in SCs, which is related to reduced protein levels of LC3, Beclin 1, ATG12, Rab5, Rubicon, and Dock180 [54]. Another study found that miR-26a reduces autophagy in swine SCs via downregulating ULK2 expression, suggesting a potential role for this microRNA in swine spermatogenesis [55]. With this in mind, non-coding RNAs (ncRNAs) are a potential target for modulating autophagy in treating male fertility, but further studies are needed to better understand their role in this condition.

### Leydig cells

The Leydig cells are located in the interstitium of the testis, and they contribute to the production of testosterone, a steroid hormone responsible for the development of the male reproductive system and the regulation of secondary sexual characteristics in men [56]. Decreased serum testosterone level is usually observed in primary or late-onset hypogonadism [57, 58].

Autophagy is known to regulate lipid metabolism in many cell types through lipophagy to access cholesterol and triglyceride sources from lipid droplets. In Leydig cells, free cholesterol, the substrate for testosterone synthesis, is released upon hormone stimulation [28]. Autophagy is involved in testosterone production via the regulation of lipid metabolism [9]. When autophagy is impaired in the Leydig cells of aging male mice, serum testosterone levels decrease – similar to late-onset hypogonadism – which can negatively affect sexual behavior. In addition, the decrease in autophagy leads to an accumulation of Na^+^/H^+^ exchanger regulatory factor 2 (NHERF2), decreasing the expression of scavenger receptor class B, type I (SR-BI), an HDL receptor. Decreased expression of SR-BI inhibits cholesterol uptake in Leydig cells and eventually leads to reduced testosterone biosynthesis [59]. Autophagy was also shown to be involved in testosterone production by degrading intracellular lipid droplets and total cholesterol in primary rat Leydig cells [60].

ncRNAs have been found to play a role in the autophagy process during spermatogenesis. Some ncRNAs have been reported to impose an inhibitory effect on autophagy in the male reproductive system, while others have shown a stimulatory effect. For example, the microRNA miR-200a is upregulated in patients with spermatogenesis disorders. ATG7 was identified as a direct binding target of this microRNA [61]. Interestingly, triptolide (TP), a diterpenoid triepoxide, can increase the expression of miR-200a in the MLTC-1 Leydig cell line and suppress autophagy in these cells. Additionally, activation of ATG7 using miR-200a antagomir can protect Leydig cells against TP-induced suppression of autophagy [61].

## Autophagy and aging-related conditions

Aging is a biological phenomenon that leads to a gradual deterioration of cellular functions and eventually to the death of the organism [62]. This occurs as an organism accumulates harmful changes during its lifetime, to which it responds with defense, repair, and maintenance mechanisms [63]. The World Health Organization (WHO) estimates that in 2016, 933.50 million (of which 431.36 million were men) of the total world population belonged to the aging population (≥ 60 years of age), and that this number is expected to increase 3.25-fold to 3038.77 million by 2060 [64]. Aging and increasing life expectancy in men are associated with various chronic diseases, including reproductive disorders that threaten life or affect the quality of life [65]. Since autophagy plays an important role in cellular homeostasis and organ health, including protein/organelle quality control, regulation of lipid metabolism, adaptation to starvation, anti-aging and anti-degeneration, development, immune response, programmed cell death, and tumor suppression [5, 25, 66], its dysfunction is associated with many of the diseases mentioned above [67, 68]. These disorders are typically characterized by the accumulation of damaged proteins or organelles in conjunction with decreased autophagy [68–70], as described in more detail below.

### Male infertility

#### Aging and cellular senescence

Aging is an inevitable biological process and an important risk factor for many diseases. A prevalent hypothesis speculates that aging is mostly driven by cellular senescence (CS), a cellular process in which the cells’ cycle is arrested, and several distinctive alterations appear in affected cells [71]. CS can be initiated and fueled by various intrinsic and extrinsic triggers. Mitochondrial damage, oxidative stress, the accumulation of irreparable genetic mutations, telomere attrition, and increased expression of cell cycle inhibitors like p16^INK4A^ and p21^CIP1^ are among the most important CS inducers [72].

It has been shown that autophagy and CS can have common stimuli, including oxidative stress, organelle stress, telomere attrition, and DNA damage. In this concept, autophagy has an anti-senescence function and is increased in senescent cells to remove dysfunctional macromolecules or organelles. However, an emerging hypothesis suggests that autophagy facilitates the synthesis of senescence-associated agents and promotes CS [73]. Early evidence showed that the knockdown of ATG7 or ATG5 genes can suppress the autophagy process, which in turn increases the cellular levels of ROS and promotes CS in human fibroblasts [74]. Autophagy can also keep homeostasis under stressful situations and suppress CS. The sirtuin 1 (SIRT1)-dependent signaling is one of the pathways that shown could be activated in stressful conditions and induces autophagic activity [75].

Mitochondrial dysfunction plays a critical role in CS [76, 77]. This condition can increase the generation of mitochondrial superoxide related to CS and aging. A distinctive type of autophagy called mitophagy can be activated to remove dysfunctional mitochondria from senescent cells. Such mitochondria are marked by parkin, which induces ubiquitination of outer membrane proteins and interacts with LC3 to recruit autophagosomal membranes to the targeted mitochondria [78]. The first report on the paradoxical relationship between autophagy and CS showed that the inhibition of autophagy regulators could also suppress oncogene-induced senescence [79].

Further investigations revealed that a particular type of autophagy called the TOR-autophagy spatial coupling compartment (TASCC) actively participates in amino acid recycling for the massive synthesis of secretory proteins during CS [80]. Subsequent studies supported the stimulatory effect of autophagy on CS. For example, not limited to the metabolic support for senescent cells, the autophagy system can accelerate the degradation of nuclear lamin in mammalian cells. The autophagy proteins LC3/Atg8 have been shown to interact directly with the nuclear lamina protein lamin B1. This interaction leads the complex to the cytoplasm and delivers lamin B1 to the lysosome. In oncogenic situations like activation of RAS, the nuclear envelope integrity declines, and autophagy suppresses tumorigenesis [81]. Proteolytic degradation of histones for remodeling chromatin fragments is another hallmark of senescent cells, which was shown to be mediated by autophagy [82]. The presented findings reveal the complex nature of autophagy in CS regulation that necessitates further investigations to shed light on unknown aspects of this interaction.

It is believed that aging affects the normal spermatogenesis and is associated with fertility decline. Various DNA damages, such as microdeletions in Y-chromosomes, changes in telomere length, and accumulation of point mutations, have been reported in aged spermatogenic cells, while the general capacity of the DNA repair system diminished [83]. Testicular cells of older men have also been shown to have different epigenetic patterns, as well as different miRNA and long non-coding RNA (lncRNA) expression levels [84]. It is revealed that spermatogonial stem cells (SSCs) have shorter telomeres and proliferate more actively compared to younger SSCs. However, SSCs gradually lose sperm-forming potential because the higher proliferation rate of aged SSCs attributed to the hyperactivation of the Wnt7b-JNK signaling pathway, that in turn alters the metabolic state of these cells and induces glycolysis [85]. In addition to the aforementioned molecular signatures of aged spermatogenic cells, the dynamics of the germ cell population can be affected by aging. For instance, Sertoli cells of men older than the mid-40s undergo morphological changes, increasing their nuclear and nucleolar size. Malfunction/exhaustion of Sertoli cells is thought to lead to a decline of spermatogenic efficiency and elevated numbers of proliferating A-dark spermatogonial in older men [86]. These alterations extend beyond the mentioned testicular cells. Peritubular cell in the human testes is another cell type involved in sperm transportation, SSCs niche, and immune-surveillance. These cells develop a state known as “replicative senescence” in the aging human testis, including dramatic cellular morphology changes and impaired proteostasis [87].

Based on what we have discussed so far, aging and CS have a negative impact on spermatogenic cells. Furthermore, autophagy is known to play a vital function in CS. These findings support the idea that autophagy lies at the crossroads of age-related reproductive complications. However, there is an absolute need for studies on autophagic changes in CS of reproductive tissues.

#### Inflammaging

Inflammaging, defined as chronic, low-grade systemic inflammation, appears to be a common factor responsible for the development of disease in aging men [88]. With age, both innate and adaptive immunity undergo fundamental changes. Age-related changes in the immune system lead to increased levels of inflammatory mediators such as interleukin (IL)-1, IL-6, and tumor necrosis factor α (TNFα), along with an increase in cellular debris and damage-associated molecular patterns (DAMPs), senescent cells, Inflamma-miRs, components of the coagulation pathway, agalactosylated N-glycans, meta-inflammation; decrease in proteasome disposal capacity and autophagy, dysbiosis of gut microbiota and impaired complement regulation contributing to inflammaging [89]. Many aging-related diseases (ARDs), such as obesity, type II diabetes, prostatitis, cardiovascular disease, and Alzheimer’s disease, share this common pathogenesis mechanism [89–91]. The male gonads and reproductive tract are examples of tissues that inflammation may affect. Since the male reproductive tract and accessory glands are crucial for sperm maturation and final semen composition, any damage due to inflammation in the male reproductive tract can lead to subfertility or even infertility [88, 92].

**Fig. 4:**
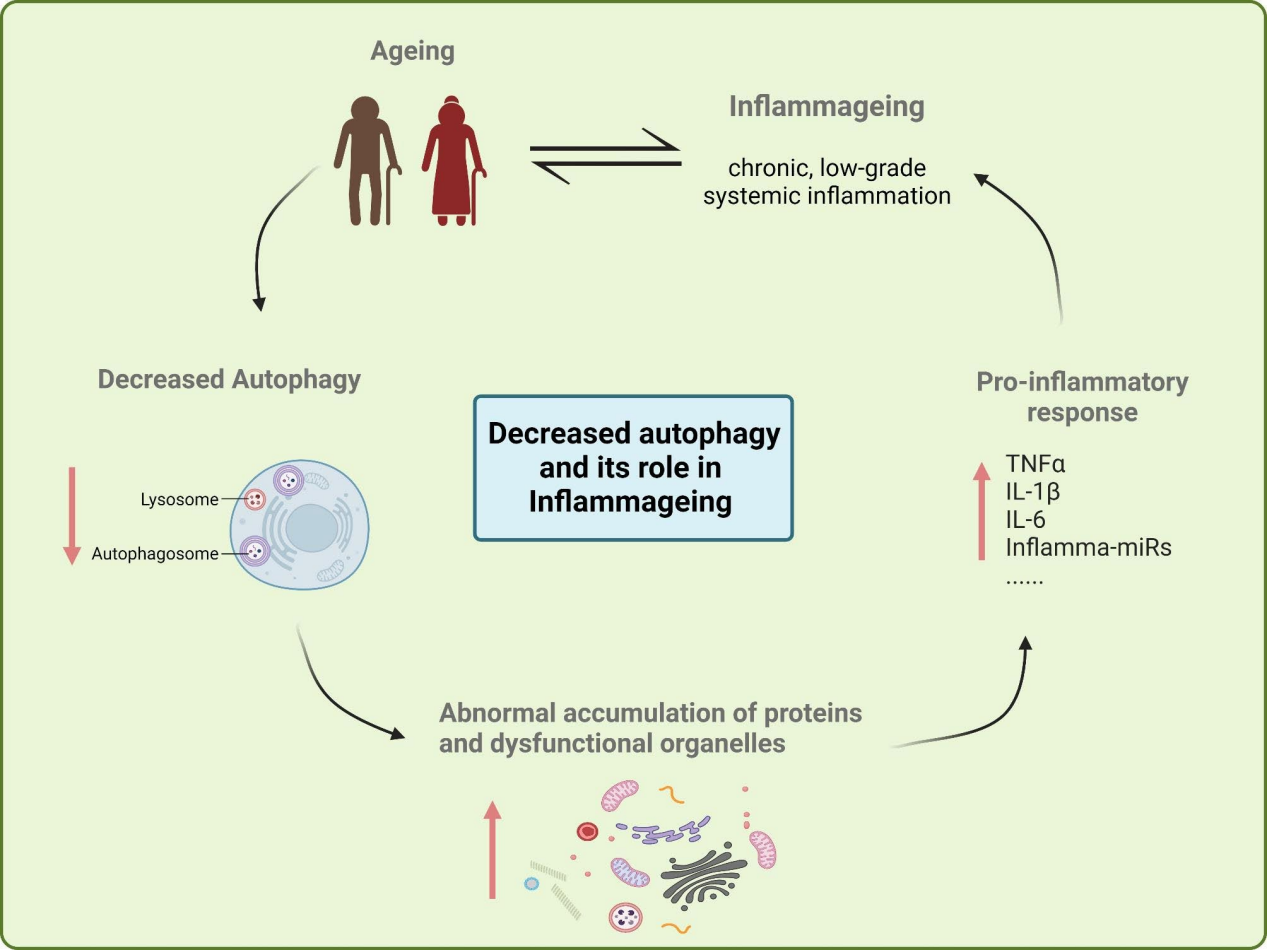
The role of aging-related decline of autophagy in Inflammaging. The aging process leads to a decrease in autophagy, resulting in the abnormal accumulation of proteins and dysfunctional organelles (especially mitochondria), which is one of the mechanisms that lead to Inflammaging. TNFα: tumor necrosis factor α; IL: interleukin. ↑: increase or induction; ↓: decrease or inhibition

Autophagy is known to have a significant impact on the induction and modulation of inflammatory responses in the male reproductive tract. Understanding the balance between autophagy and inflammation would therefore be a promising starting point for the development of more efficient therapeutic interventions [88, 92–94]. The aging process leads to a decrease in autophagic capacity, resulting in the abnormal aggregation of proteins and accumulation of dysfunctional organelles (especially mitochondria), which supports the notion that inflammaging can be triggered by the aggregation of cellular elements that are not properly eliminated (Fig. 4). The accumulation of poorly recycled organelles leads to an increase in inflammatory mediators and hence induces an inflammatory response which accelerates the development of ARDs [89, 95, 96]. This may also be the case in the pathophysiological processes associated with inflammaging in the male reproductive tract.

#### Obesity and diabetes

Metabolic syndrome (MetS) is a term used to describe several health issues like obesity, diabetes, dyslipidemia, and hypertension. The onset of MetS has become one of the key world health challenges in recent years. The prevalence of MetS is strongly geography-dependent. Nevertheless, the prevalence rate of MetS seems to increase with aging [97]. It was shown that the prevalence of obesity among males aged 65 to 74 increased from approximately 10% in 1999–2002 to 41.5% in 2007–2010 [98]. Obesity is now known to have deleterious effects on male fertility potential [99–103]. Epigenetic and hormonal alterations, scrotal hyperthermia, increased ROS levels, and lower sperm quality (lower total count, motility, and DNA integrity) are believed to be the primary causes of infertility in males with obesity [99, 102–105]. Autophagy is now established to be a key component in the pathophysiology of this situation [103]. Indeed, some studies have found activation autophagy in individuals with obesity [103, 106, 107]. Obesity may alter autophagy via dysregulated expression of associated genes and activation of proinflammatory cytokines [108, 109]. Previously, we found that mRNA levels of autophagy-related genes such as ULK1, Beclin 1, and Bcl-2 were up-regulated in semen samples from individuals with obesity compared to normal-weight individuals [110]. It has also been shown that autophagy is upregulated in mice with impaired spermatogenesis induced by a high-fat diet [103]. Inhibition of autophagy by intraperitoneal injection of chloroquine (CQ) improved sperm count, viability, and motility. In addition, CQ, combined with 3-methyl adenine (3-MA), another inhibitor of autophagy, improved spermatogenesis in vivo [103]. Overall, the current evidence suggests that disturbances in autophagy, either by overactivation or downregulation, may lead to infertility, especially in males with obesity.

Several studies have shown evidence of a negative relationship between diabetes and male fertility potential. For instance, it has been shown that individuals with type 2 diabetes mellitus (T2DM) have a reduced progressive motility of sperm and an increased sperm DNA fragmentation compared to non-diabetic patients [111]. Additional comparative clinical research has similarly shown that individuals with diabetes have reduced sperm concentration, lower total sperm count, and even decreased ejaculate volumes [112, 113]. As previously mentioned, dysregulated autophagy has been discovered in several aging-related diseases, including diabetes [114]. Moreover, the overnutrition of genetically modified mouse models carrying Atg7 (+/-) and Atg4b genes deficiency with a high-fat diet or sugar-enriched drinking water could induce insulin resistance via accelerating lipid accumulation in their adipose and non-adipose tissues [115, 116]. These findings confirmed that autophagy is necessary to counteract metabolic stress, and deleterious genetic changes in autophagic-related genes can be considered risk factors for MetS diseases such as T2D [115, 116]. The negative effects of diabetes-induced autophagy dysregulation on the male reproductive system have also been reported in animal models [117, 118]. For example, one study has proposed a potential link between diabetic hyperglycemia and the dysregulation of autophagy in the epididymal tissues of rat models. This dysregulation may contribute to the development of diabetes-induced epididymal damage [118]. It should be noted that the potential impact of diabetes on male reproductive system has been attributed to the interaction between ROS and autophagy in the testes, mediated via the PI3K/Akt/mTOR signaling pathway [119]. According to the evidence, the deregulation of autophagy, especially the attenuation of autophagy capacity, is associated with MetS diseases and may proceed to fertility dysfunctions and overall health problems in elderly men.

#### Testicular toxicity (chemotherapeutics and other compounds)

Multiple in vitro and in vivo studies imply that drugs or chemicals that influence autophagy may affect male fertility. As we know, aging is among the main risk factors for cancer [120]. For years, chemotherapy stands at the first line of cancer therapy strategies. Chemotherapy agents work non-specifically on the whole body and are effective in the treatment of metastatic cancers. However, this approach causes severe side effects as it can also act against normal rapid-dividing cells [121–124]. Oligospermia, azoospermia, and infertility are the most prevalent side effects reported in mouse tumor models and humans under chemotherapy treatments [125, 126]. To what degree the fertility ability of patients is damaged depends on the received therapeutic regimen, including the type of chemotherapy agent, applied dose, and individual differences [127]. Meanwhile, the side effects of chemotherapy can be even worse in older patients. The differentiation of spermatogonia is the most sensitive phase of spermatogenesis to chemotherapy agents [128].

Some chemotherapy agents, such as cyclophosphamide (CP) and cisplatin, severely damage the telomeres of SSCs. They could also inhibit telomerase activity by suppressing the expression of telomerase reverse transcriptase (Tert) and telomerase RNA component (Terc) [129]. Such hazardous consequences not only threaten the self-renewal property of SSCs but may convey to and negatively affect the next generation. Beyond germ line cells, chemotherapy can damage somatic cells of the spermatogenesis micro-environment. For example, the administration of doxorubicin (Dox) in rats decreased the production of transferrin in the seminiferous epithelium [130]. The glycoprotein transferrin, secreted by Sertoli cells, plays an important role in regulating spermatogenesis [131]. Lack of transferrin upon Dox administration induces tangible functional and morphological damage to Sertoli cells, which spoils the supportive contribution of these cells during the differentiation of SSCs [130]. There are also reports on the adverse effect of chemotherapy on other cells like Spermatocytes and Leydig cells [132, 133].

New findings have suggested that chemotherapy-induced side effects can occur due to the dysregulation of autophagy [134, 135]. For example, it is documented that Dox quenches the expression of GATA4 and S6K1 genes that actively regulate the expression of several critical autophagy genes, such as Atg12, Atg5, Beclin 1, and Bcl-2 [136]. LC3-II is another autophagy-associated protein whose expression is influenced by chemotherapy agents. The expression level of LC3-II in hepatocytes of patients who received etoposide seems to increase significantly. LC3-II participates in the activation of p53 and AMPK, which in turn promotes the activation of autophagy [137]. However, the autophagy-stimulating effect of chemotherapy agents is not universal. Some studies implicate that autophagy can be suppressed upon the application of chemotherapy agents. This paradoxical effect on autophagy following chemotherapy treatments is greatly tissue- and drug-dependent and is comprehensible considering the dual function of autophagy in inducing cell death or promoting cell survival [138]. Based on the facts, chemotherapy can potentially affect autophagy in many tissues, but there is still a long way to go until we completely understand the changes in autophagy in testicular tissue and sperm samples following chemotherapy.

Toxicological studies have also uncovered several chemicals that impair autophagy in male reproductive cells. For example, 4′-Nonylphenol (4-NP) is an organic cytotoxic chemical proven to reduce rat SCs’ viability [139]. This chemical was shown to induce apoptosis, autophagy, and necrosis all at once, providing insight into the mechanism by which it exerts its effects. After considering the evidence, the authors concluded that inducing autophagy may be the survival mechanism to counteract apoptotic cell death. They further suggested that ROS-dependent JNK- and Akt/AMPK/mTOR pathways may be involved in the process of 4-NP-induced cytotoxicity [139]. A study investigating the synergistic cytotoxic effects of Tebuconazole and Econazole (MIX) on a mouse Sertoli cell line (TM4 cells) showed that autophagy can protect male reproductive organs from toxins [140]. Both autophagy and apoptosis were elevated in TM4 cells after exposure to MIX. It was also shown that energy stress and subsequent activation of the AMPK-ULK1 pathway led to the activation of autophagy in this scenario. Furthermore, bafilomycin A1 (an autophagy inhibitor) treatment resulted in increased apoptosis [140]. In light of these results, the authors concluded that elevated autophagy in the situation is not a death mechanism but rather an adaptive cytoprotective response [140].

As can be seen from various investigations, differing conclusions have been reached about whether and to what degree activation of autophagy in certain pathological situations is regarded as cytoprotective or pathologic [139, 141, 142]. These studies may have methodological differences, but they all share the finding that autophagy plays a role in various pathophysiological conditions affecting the male reproductive system.

### Erectile dysfunction

Sexual dysfunction is not only a common concomitant of chronic diseases or their therapies but is also considered a side effect of chronic diseases [143, 144]. Statistics from the US National Health and Nutrition Examination Survey (NHANES) show that the prevalence of erectile dysfunction (ED) increases with age. Among men aged 20–39 years, the prevalence of ED is approximately 5%. In men aged 40–59 years, it increases to 14.8%, and in men aged 70 years and older, up to 70% are affected [145, 146]. The disorder is particularly common in the elderly, men with obesity, and diabetic patients [145–148]. Lifestyle modification and weight loss have been shown to improve ED [147]. ED is correlated with obesity-related dyslipidemia, which can be treated with a healthy diet, regular exercise, and medications [149]. Obesity is well known to be associated with autophagy dysregulation, and activation or inhibition of autophagy is tissue-specific in the situation [108]. Hyperlipidemia also impaired erectile function in rat models by inducing cavernosal fibrosis, apoptosis, and suppressing autophagy [150]. Another study found that long-term 5α-reductase inhibitors (5ARIs) therapy reduces erectile function in aging rat models through decreasing autophagy and increasing apoptosis in cavernous smooth muscle cells, confirming the significance of autophagy in proper erectile function [151]. In the case of diabetes, mice models with type 2 diabetes mellitus-induced ED were shown to have much lower levels of the miR-301a-3p in their corpus cavernosum compared to the normal group [152]. Later, it was shown that treating hypoxia-induced ED rat models with miR-301a-3p-enriched exosomes might enhance erectile function due to its autophagy boosting and apoptosis inhibiting properties [153]. This supports a role for autophagy in ED, while further studies warranted to identify the precise mechanisms of pathogenesis.

### Prostate cancer and benign prostatic hyperplasia

In older men, reproductive health complications such as cancer and hyperplastic enlargement of the prostate are far more prevalent [154, 155]. Benign prostatic hyperplasia (BPH), is the most common benign condition in older men and develops from growths of the prostatic transitional zone around the urethra. Lower urinary tract symptoms (LUTS) caused by BPH affect up to 50% of males over 50 [156]. Medication is recommended for mild cases of BPH, and surgery is recommended for severe cases or patients poorly responding to medication [156]. Men with BPH are at higher risk for comorbidities such as MetS and ED, which negatively affect male fertility [157, 158]. Autophagy may have a role in BPH since androgen deprivation induces autophagy in PWR-1E prostate epithelial cells, and inhibiting autophagy using 3-MA dramatically increases the apoptosis rate of these cells [159]. Another study found that the long-term use of 5‐ARI can reduce insulin-like growth factor 1 (IGF‐1) expression in prostate fibroblasts and induce autophagy in the epithelial cells of the prostate transitional zone [160]. Further evidence about the role of autophagy in the pathophysiology of BPH comes from the study that has found decreased autophagy flux in BPH-1 cell lines [161].

On the other hand, PCa is the most prevalent non-skin cancer identified in males, ranging from a localized, indolent progression to a quickly developing, metastatic malignancy [162]. Untreated PCa patients are predicted to have a high mortality rate due to the increased risk of metastasis, such as liver metastases [163]. Ethnicity, age, family history, and IGF all have a recognized role in cancer pathogenesis. Furthermore, genetic susceptibility, androgens, and environmental factors such as diet are the most important known risk factors for PCa [164, 165]. Prostate-specific antigen (PSA) is the most important marker for screening, diagnosis, staging, and monitoring response to treatment of PCa [166, 167]. Treatment strategies are based on cancer stage and tumor characteristics, including radiation therapy, hormonal therapy, chemotherapy, surgery, and cryotherapy [168]. A reciprocal relationship between PCa and male fertility is controversial, as some experiments claim that infertile men have low androgen levels that reduce the risk of PCa [169], while others show that infertile men are at higher risk for cancer development, including PCa [170]. Reproductive cancers can negatively affect male fertility through direct and indirect pathways [168].

Autophagy has been shown to play a dual and opposing role in cancers [171]. On the one hand, autophagy is thought to help tumor cells maintain proliferation and survive in a harsh tumor microenvironment by increasing tumor cell accessibility to recycled molecules and enabling energy transfer from the tumor stroma [172, 173]. Consistent with this, inhibition of autophagy by genetic techniques or pharmaceutical products has been shown to slow tumor progression in animal studies [174]. On the other hand, there are reports that autophagy is impaired in cancer tissues and that induction of autophagy can limit cancer progression. For example, altered expression of autophagy-related genes, such as Beclin 1, has been shown to over-activate autophagy in PCa cells and inhibit tumorigenesis [175, 176]. In addition, it has been shown that several drugs with anticancer activity, such as metformin and rapamycin, exert their antiproliferative effects mainly through their stimulatory effects on autophagy [177, 178]. Autophagy is thought to limit genomic damage and prevent the accumulation of deleterious mutations in cancer cells under microenvironmental stressors [179]. Indeed, autophagy may paradoxically protect cancer cells from environmental stressors and promote their survival or, conversely, induce cancer cell death [171, 180]. The discovery of the unique involvement of ncRNAs in autophagy modulation adds to the evidence that autophagy plays a role in PCa development and progression [181]. Table 1 summarizes examples of these ncRNAs. Given the important role of this process in PCa, these autophagy-related biomarkers may have prognostic value for PCa patients [182].

**Table 1:** Examples of Autophagy regulation by non-coding RNAs in PCa

NcRNAs	In vitro/In vivo	PCa Cell line(s)	Patients/Animal model	Target pathway(s)	Effect on autophagy	Finding(s)	Ref.
LncRNA PRRT3-AS1	In vitro/In vivo	DU145, LNCaP, PC3, IA8 and IF11	BALB/c- nude mice bearing PC3 xenograft	mTOR pathway	↓	Silencing the PRRT3-AS1 was shown to trigger the PPARγ gene, which might limit PCa cell growth while also promoting apoptotic cascade and autophagy.	[181]
LncRNA SNHG12	In vitro/In vivo	22RV1, Du145, LNCaP, MDaPCa2b	Serum from PCa patients	PI3K/AKT/mTOR pathway miR-195-Cyclin E1 (CCNE1)	↓	SNHG12 upregulation increased cell survival and prevented apoptosis and autophagy in PCa cells through controlling CCNE1 expression via miR-195 sponging. The expression of SNHG12 was shown to be increased in PCa patients’ serum.	[183]
LncRNA SNHG1	In vitro/In vivo	LNCaP, PC3 and DU145	PCa tissue sample from patients / BALB/c nude mice bearing PC3 xenograft	Targeting EZH2 Wnt/β-catenin and PI3K/AKT/mTOR pathways	↑	SNHG1 is abundantly expressed in PCa. and induces PCa cell proliferation, apoptosis, and autophagy. SNHG1 expression is favorably connected to PCa patient prognosis, but EZH2 expression is adversely associated with their prognosis.	[184]
LncRNA HULC	In vitro/In vivo	DU-145, PC3, LNCaP, and RWPE-1	NOD-SCID mice bearing PC3 xenograft with aberrant HULC expression	mTOR pathway Beclin 1	↓	HULC knockdown increased PCa cell susceptibility to irradiation by boosting apoptotic cascade and autophagy while suppressing the cell cycle in vitro and in vivo.	[185]
LncRNA PCDRlnc1	In vitro/In vivo	PC3-DR and DU145-DR	Metastatic PCa tissue or blood samples from patients / Balb/c nude mice-bearing PC3-DR or PCDRlnc1-deleted PC3-DR xenograft	UHRF1-Beclin 1	↑	PCDRlnc1 enhances autophagy and resistance to docetaxel in PCa cells by interaction with UHRF1 and boosting its transcription, increasing Beclin 1 expression.	[186]
miR-205	In vitro/In vivo	DU145, and PC3	SCID mice-bearing DU145/miRVec and DU145/miR-205 xenograft	RAB27A and LAMP3	↓	miR-205 is downregulated in PCa. Loss of miR-205 potentially helps PCa cells develop mesenchymal traits while establishing a favorable autophagic environment that provides a chemoresistant phenotype. Boosting miR-205 expression may be a viable strategy for overcoming platinum compounds-resistance in PCa.	[187]
miR-34a	In vitro/In vivo	PC3 and DU145	PCa tissue samples from patients	AMPK/mTOR-ATG4B pathway	↓	miR-34a is downregulated in PCa. Overexpression of miR-34a improves chemosensitivity, decreases cell proliferation, and increases apoptosis in PCa cells.	[188]
miR-­124 and miR-­144	In vitro	DU145 and PC3	-	PIM1	↓	Hypoxia causes additional downregulation of miR-124 and miR-144. Overexpression of miR-124 or miR-144 suppresses hypoxia-induced autophagy and improves radio-sensitivity.	[189]
miR-96	In vitro/In vivo	LNCaP and 22Rv1	PCa tissue sample from patients / Athymic nude mice bearing LNCaP xenograft	mTOR pathway ATG7	↑ Biphasic ↓	miR-96 regulates hypoxia-induced autophagy, at least to some degree. Depending on miR-96 expression levels, miR-96 can either stimulate or inhibit autophagy by primarily acting on mTOR or ATG7 pathways. Up- or down-regulation of miR-96 reduced tumor growth in vivo and PCa cell proliferation in vitro.	[190]
miR-301a/b	In vitro	LNCaP, PC3 and DU145	-	NDRG2	↑	Hypoxia resulted in a substantial overexpression of miR-301a/b in PCa cells. miR-301a/b can specifically target the 3’UTR of NDRG2 and reduce its expression. Reduced expression of NDRG2 boosted autophagy and cell survival while decreasing apoptosis.	[191]

↑: increase or induction, ↓: decrease or inhibition, PCa: prostate cancer; UHRF1: ubiquitin-like with plant homeodomain and ring finger domains 1.

## Autophagy modulation as a new therapeutic approach

### Male infertility treatment

In the last several years, it has become clear that certain biomolecules may be used to treat male reproductive disorders. Animal models have been applied to study the effects of aging on male fertility and to discover new therapeutics. For instance, cordycepin (COR), an active component of the mushroom *Cordyceps militaris Linn*, was investigated for its effect on testicular dysfunction in senile rats (12-month-old). As a result of COR treatment (20 mg/kg), senile rats showed an improvement in spermatogenesis parameters. Additional findings included the upregulation of SIRT1, a histone deacetylase, and the downregulation of autophagy-related mTORC1 proteins in the COR treatment group compared to the aged-control group [192].

As we highlighted earlier, obesity is associated with male infertility [99, 102–105]. For instance, increased sperm production, viability, and motility were observed after intraperitoneal injection of CQ in obese mice on a high-fat diet, where autophagy is known to be excessive. CQ and 3-MA, another autophagy inhibitor, further enhanced spermatogenesis in vivo [103]. This can be explained because excessive autophagy ultimately results in cell death [193]. Therefore, it seems reasonable that a therapy strategy for excessive activation of this mechanism would include reducing it to a normal level. As mentioned earlier, the metabolic disease diabetes is also known to negatively affect male reproductive health [194]. Insulin therapy improves fertility in rat models of diabetic hyperglycemia by correcting dysregulated autophagy in epididymal tissue [118]. This improved autophagy can be attributed to the reduction of oxidative stress-induced damage to the epididymis following insulin treatment [118]. Lycium barbarum polysaccharide (LBP), a dietary supplement often utilized in Chinese medicine, has been shown to ameliorate diabetic testicular dysfunction by modulating excessive autophagy in mice. Gene and protein expression analysis revealed that after LBP treatment, autophagy marker proteins such as LC3I were downregulated in testicular tissue. It was also discovered that LBP negatively regulates autophagy by activating the PI3/Akt pathway [195].

Chemotherapy is often the first line of cancer therapy [196–199]. It has long been known that these drugs can interfere with testicular function and spermatogenesis [200]. Based on this fact, different animal models have been used in studies to identify autophagy-based therapies for these chemotherapy-induced side effects [201–203]. For instance, moderate induction of autophagy using rapamycin was shown to protect mouse spermatogonial progenitor cells (SPCs) from the cytotoxic effects of busulfan. However, exposure to high levels of rapamycin (10 μm to 50 µM) exacerbated SPCs death under busulfan stress, indicating autophagy has a dual role in controlling SPCs fate [201]. In another study, Korean red ginseng (KRG) extract was used to attenuate Dox-induced testicular dysfunction in Sprague-Dawley rats, and these effects were discovered to be achieved through the modulation of inflammatory, oxidative, and autophagic responses. To counteract the effects of Dox on autophagy, results revealed that nine weeks of daily oral administration of KRG significantly reduced mRNA and protein levels of mTORC1 [202].

Furthermore, L-carnitine therapy (2.1 ml/kg daily for five days, orally) has been demonstrated to improve testosterone levels, sperm motility, and viability and reduce apoptosis of germ cells in rat models of CP-induced testicular dysfunction. It was also found that L-carnitine administration leads to up-regulation of protein and mRNA levels of LC3 and Beclin 1 leading to autophagy modulation compared to the CP group [203]. As can be seen from Table 2, it has also been found that modulating autophagy in various models of testicular toxicity can be a potential option for treatment or at least prevention. Based on these findings, regulating autophagy as a therapeutic option for male reproductive complications caused by chemotherapy merits further investigation.

**Table 2:** Examples of pharmacological compounds or therapeutical procedures found to improve male fertility in different toxicity models by modulating the autophagy process

Drug/ compound/ procedure	Toxicity model	Cell line	Animal model	Target pathway(s)	Effect on autophagy	Finding(s)	Ref(s)
Rapamycin	ZEA cytotoxicity	Rat Leydig cells	-	mTOR pathway (well-known target of Rapamycin)	↑	At various dosages, ZEA effectively decreased Leydig cell proliferation by triggering apoptosis. Autophagy induction using rapamycin reduced apoptosis and protected rat Leydig cells from ZEA cytotoxicity.	[141]
Naringenin	Cd toxicity	-	Rat	-	↓	Cd toxicity can result in pathological changes in rat testicular histopathology, oxidative stress (OS), and marked decreases in serum concentrations of GnRH, FSH, LH, and testosterone. Cd toxicity also leads to the over-expression of autophagy-related proteins, P62 and LC3 II, in the testis. Administration of flavonoid naringenin (50 mg/kg, orally) was shown to protect rat testicular tissue against cadmium-induced damage, at least partly through suppressing Cd-induced autophagy.	[142]
SIP	Acrolein-induced cytotoxicity	Mouse Leydig cells	-	p38 MAPK and PI3K/Akt pathway	↓	SIP was shown to alleviate acrolein-induced cytotoxicity in mouse Leydig cells by suppressing autophagy and apoptosis, further confirming the opposing dual functions of autophagy	[204]
Resveratrol	Nicotine-induced oxidative damage	Mouse TM3 Leydig cell	-	AMPK phosphorylation and suppressing mTOR pathway	↓	Resveratrol treatment prevented nicotine-induced damage to Leydig cells by inducing autophagy through AMPK phosphorylation and suppressing the mTOR pathway.	[205]

↑: increase or induction; ↓: decrease or inhibition; OS: oxidative stress; ZEA: Zearalenone; Cd: Cadmium; SIP: Squid ink polysaccharide.

### Erectile dysfunction treatment

**Fig. 5:**
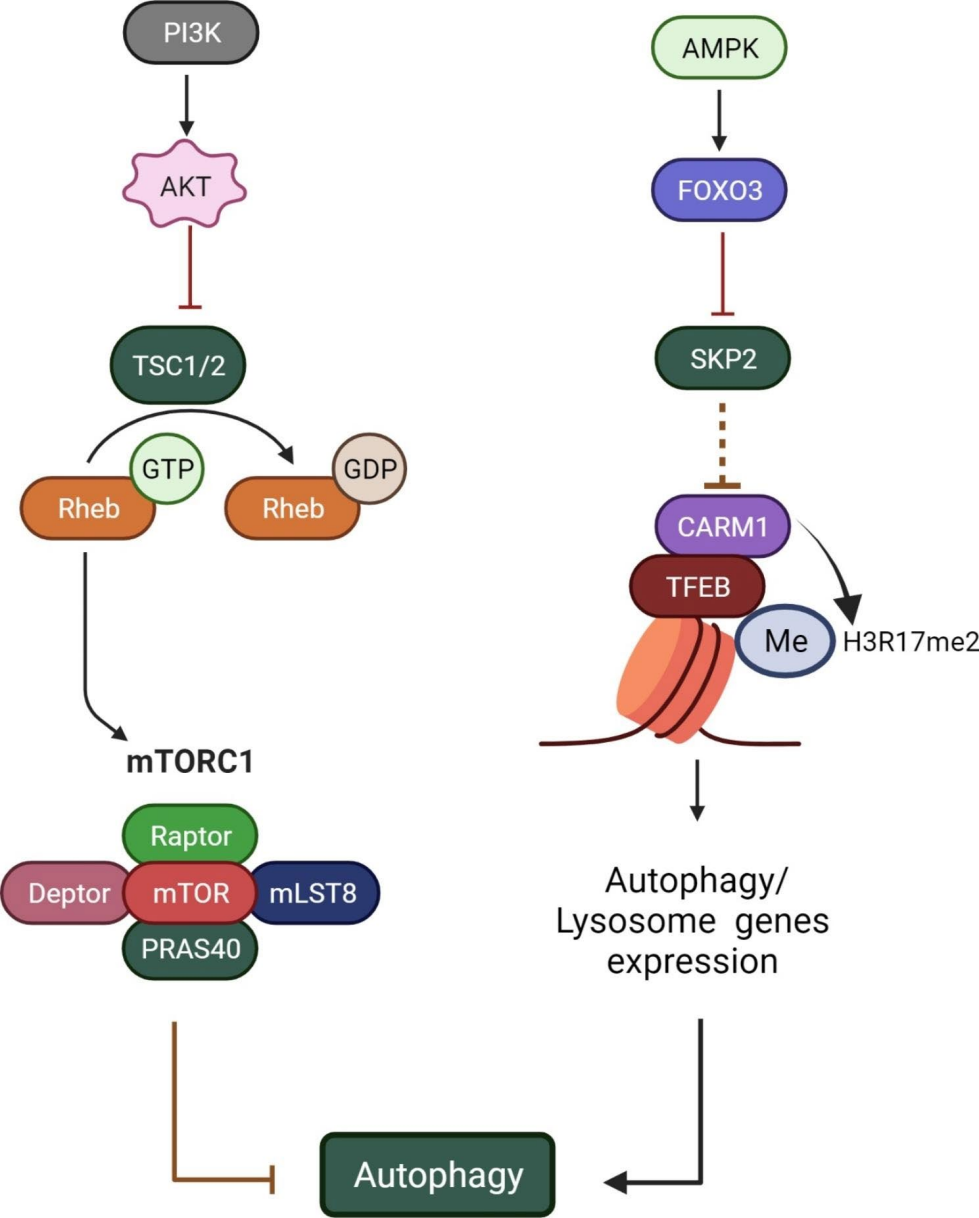
Examples of the target signaling pathways that have been studied for the treatment of ED and/or PCa. ED: erectile dysfunction; PCa: prostate cancer; PI3K: phosphoinositide 3-kinase; TSC1/2: tuberous sclerosis proteins 1 and 2; GTP: guanosine-5’-triphosphate; Rheb: Ras homolog enriched in the brain; mTORC1: mammalian target of rapamycin complex 1; AMPK: AMP-activated protein kinase; FoxO3: forkhead box O-3; SKP2: S-phase kinase-associated protein 2; CARM1: Coactivator-associated arginine methyltransferase 1; TFEB: Transcription Factor EB

Several compounds and pharmacological products that can modulate autophagy have been found to improve ED (Table 3). For example, liraglutide, probucol, and icariside II have been shown to improve ED in rat models of diabetes mellitus in various in vitro and in vivo studies [206–208]. The HMG-CoA reductase inhibitor and AMPK agonist simvastatin have been demonstrated to ameliorate diabetes mellitus-induced erectile dysfunction (DMED) in rat models by increasing autophagy through activation of AMPK-SKP2-CARM1 pathway [209]. Autophagy and Tankyrase 1 were reported to be reduced in corpus cavernosum smooth muscle cells (CSMCs) of aging rat models with ED. It was shown that promoting autophagy in CSMCs through Tankyrase 1 overexpression led to increased proliferation of these cells [210]. Transplantation of mesenchymal stem cells (MSCs) has been studied as a potential therapy for ED in experimental animals. However, oxidative stress is prevalent in DMED, which might reduce the survival of transplanted MSCs by triggering apoptosis. Based on this fact, promoting autophagy has been demonstrated to decrease apoptosis, leading to increased MSC viability and enhanced MSC-based treatment effectiveness for DMED [211]. Icariside II, in combination with metformin, was also shown to ameliorate DMED in a rat model. Besides reducing oxidative stress, it was shown that icariside II partially exerts its impact by reducing excess mitochondrial autophagy in CSMCs through the PI3K-AKT-mTOR pathway [208]. The reason for these contradictory observations is unclear. However, it suggests that excessive or insufficient levels of autophagy may lead to pathological conditions in male reproductive function and that modifying this process may be the key to finding treatments (see Table 3, and Fig. 5).

**Table 3:** Examples of pharmacological compounds or therapeutical procedures found to improve erectile function by modulating the autophagy process

Drug/ compound/ procedure	In vitro/In vivo	Cell line	Animal model	Target pathway(s) affecting autophagy	Effect on autophagy	Finding(s)	Ref(s)
Liraglutide	In vitro/In vivo	CSMC	DMED rats	-	↑	Protected against ED through regulating smooth muscle dysfunction, oxidative stress, and autophagy, irrespective of its glucose-lowering action.	[206]
Probucol	In vivo	-	DMED rats	-	↑	Improved MSCs therapeutic effectiveness by extending their survival period, increasing the antioxidant ability of MSCs, boosting autophagy, and hindering MSCs apoptosis by the Nrf2 pathway.	[207]
Simvastatin	In vivo	-	DMED rats	Activating AMPK-SKP2-CARM1 pathway	↑	Enhanced protective autophagy, improved erectile function, and alleviated Corpus cavernosum fibrosis.	[209]
Rapamycin	In vivo	-	DMED rats	Lowering the expression of AKT/mTOR and AMPK/mTOR pathways	↑	Enhanced erectile function, most likely via boosting autophagy, decreasing apoptosis and fibrotic activities, and improving endothelial function.	[212]
Tankyrase 1 overexpression	In vitro	CSMCs from aging ED rats	-	Enhancing mTOR signaling pathway	↑	Increased proliferation, and enhanced autophagy in CSMCs	[210]
Human tissue Kallikrein 1	In vivo	-	Aging ED rats	Inhibiting the PI3K/AKT/mTOR pathway	↑	Partially restored erectile function in aging transgenic rats by upregulating the protecting autophagy.	[213]
MSCT + DL-ESWT	In vivo	-	DMED rats	PI3K/AKT/mTOR pathway	↑	Improved ED, enhanced ICP/MAP ratio, decreased apoptosis, and stimulated autophagy in corpus cavernosum	[214]
USCT	In vitro/In vivo	CCECs treated with AGEs	DMED rats	-	↑	Improved ED, upregulated autophagic activity, and ameliorated cavernosal endothelial dysfunction,	[215]
Icariside II	In vivo	-	DMED rats	Enhancing PI3K/AKT/mTOR pathway	↓	Improved erectile function through decreased excess CCSMCs, mitochondrial autophagy, oxidative stress, and RAGE.	[208]
				mTOR signaling pathway		Improved diabetic ED, upregulated SMC proliferation, and the NO–cGMP pathway- Downregulated AGEs, and autophagy.	[216]

↑: increase or induction; ↓: decrease or inhibition; CSMC: corpus cavernosum smooth muscle cells; DMED: diabetes mellitus erectile dysfunction; MSC: mesenchymal stem cell; MSCT: mesenchymal stem cell therapy; DL-ESWT: defocused low-energy shock wave therapy; ICP: intracavernous pressure; MAP: mean arterial blood pressure; ED: erectile dysfunction; USCT: urine-derived stem cells transplantation; CCECs: corpus cavernosal vascular endothelial cells; AGEs: advanced glycation end products; RAGE: receptor for AGEs; SMC: smooth muscle cell.

### Prostate cancer treatment

Since dysregulation of autophagy is closely linked to PCa, autophagy modulators are currently being tested for cancer treatment [217, 218]. For example, 10- to 14-day treatment with valproic acid, an inhibitor of class I histone deacetylases, is known to stimulate autophagy by activating caspase-2 and caspase-3, thereby reducing PCa proliferation in vitro [219]. In vivo, long-term treatment with valproic acid significantly reduced tumor xenograft growth compared to untreated controls [219]. Further experiments showed valproic acid promoted autophagy by inhibiting the Akt/mTOR pathway [220]. In addition to suppressing PCa proliferation, treatment with valproic acid is also known to upregulate *NDRG1*, a metastasis suppressor, in metastatic PCa cells [221].

**Fig. 6:**
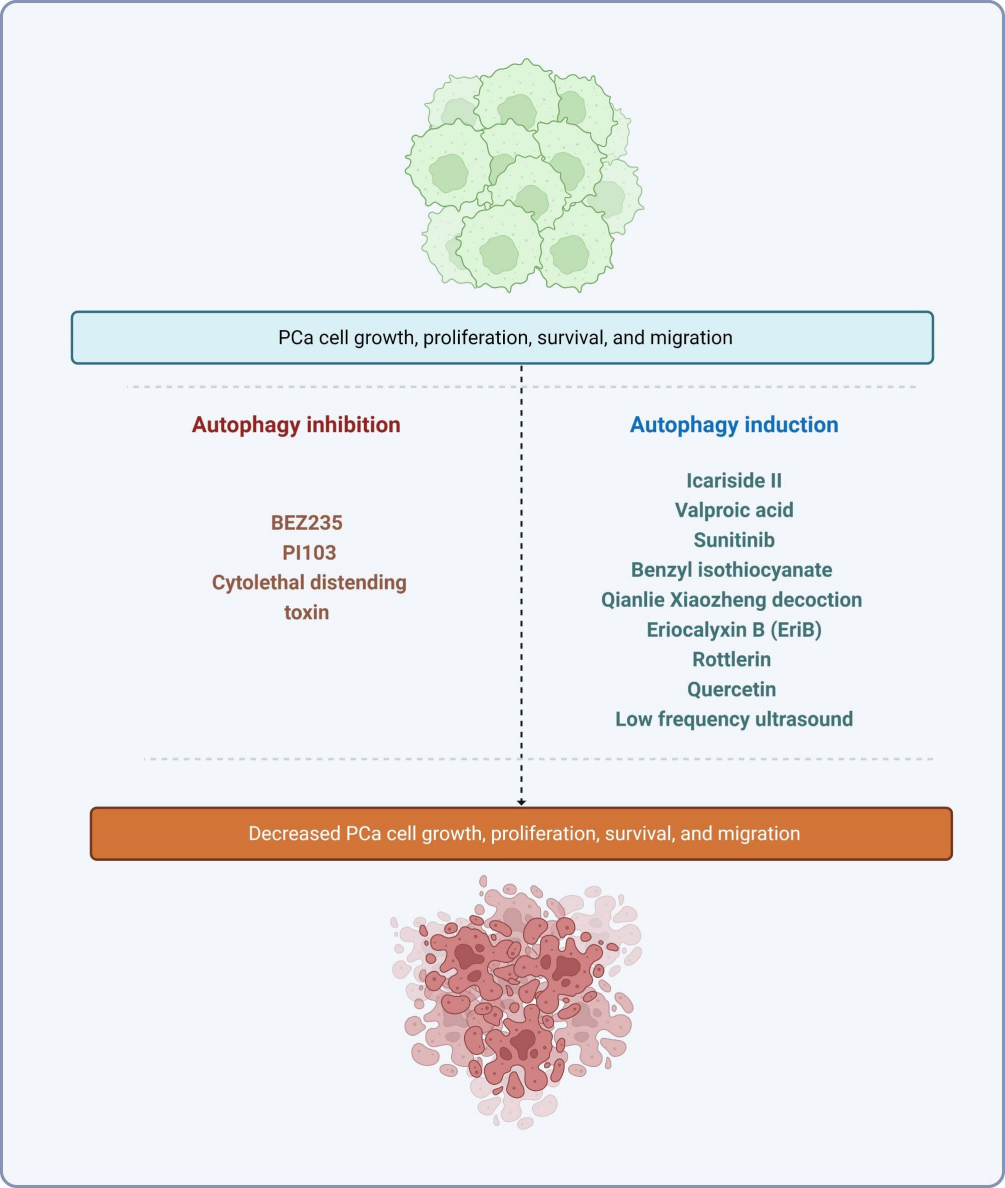
Examples of pharmacological drugs or therapeutical techniques researched for the treatment of PCa that primarily act through the Akt/mTOR pathway. As previously stated, autophagy is a double-edged sword because its over- or under-activation may result in cancer cell death, reduced survival, and migration. This fact has given rise to several studies on various PCa treatments. PCa: prostate cancer

Several other drugs have been reported to regulate autophagy in PCa cell lines and are potential targets for cancer therapies (see Table 4, and Fig. 6). These include Icariside II [222], which suppresses PCa cell proliferation, invasion, and migration in vitro by increasing autophagic activity via the PI3K/Akt/mTOR pathway. In addition, the use of benzyl isothiocyanate to treat PCa cells was shown to reduce mTOR activity, stimulate autophagy, and promote cancer cell death [223]. However, this effect can be reversed by administrating caspase inhibitors in vitro [223]. Similarly, reduced cell viability and tumor volume were observed in mice with human PCa cell xenografts treated with Qianlie Xiaozheng decoction, a traditional Chinese medicine, for 14 days, which was associated with phosphorylation of Akt and mTOR, and increased autophagic cell death [224]. Another promising anticancer agent, eriocalyxin B, also induces apoptosis and autophagy by inhibiting the Akt/mTOR signaling pathway [225].

The Akt/mTOR pathway appears to be the major signaling pathway targeted by autophagy-regulating anticancer agents. Neuregulin, for example, stimulates autophagy and simultaneously activates Akt and S6K. Another study also found that neuregulin promotes autophagy and cell death in a ROS-dependent manner by regulating signaling pathways that activate JNK and Beclin 1 [226]. Nevertheless, as autophagy plays a dual role in tumorigenesis, maintaining the balance between the cancer-promoting and cancer-suppressing functions of autophagy remains a challenge in cancer treatment and requires further investigation.

**Table 4:** Examples of the pharmacological compounds or therapeutical procedures found to improve PCa in human cell lines by modulating the autophagy process

Drug/ compound/ procedure	In vitro/In vivo	Cell line(s)	Animal model	Target pathway(s) affecting autophagy	Effect on autophagy	Finding(s)	Ref(s)
Icariside II	In vitro	DU145	-	Modulating the PI3K/AKT/mTOR pathway	↑	Suppressed human prostate tumor cell proliferation and migration	[222]
Valproic acid	In vitro	PC3, DU145 and LNCaP	-	Inhibiting the Akt/mTOR pathway	↑	Suppressed the growth of PCa cells	[220]
Sunitinib	In vitro	PC3 and LNCaP	-	Activating the ERK1/2 pathway- Inhibiting the mTOR/p70S6K pathway	↑	Inhibited PCa cell growth and induced autophagy- Increased apoptotic, and autophagic cell death	[227]
Efavirenz (EFV) and SPV122.2 (Reverse transcriptase inhibitors)	In vitro	PC3 and LNCaP	-	-	↑	Decreased proliferation - Induced genome damage- Increased autophagy	[228]
Benzyl isothiocyanate (BITC)	In vitro	Rv1, and PC3	-	Inhibiting the mTOR pathway	↑	Induced apoptosis and autophagy in human PCa cells	[223]
Qianlie Xiaozheng decoction	In vitro/In vivo	PC3	Nude mice bearing PC3 xenografts	Inhibiting the Akt/mTOR pathway	↑	Accelerated cell death in PCa cells-Inhibited tumor growth in vivo in PC3 cell line	[224]
Eriocalyxin B (EriB)	In vitro	PC3, and 22RV1	-	Inhibiting the Akt/mTOR pathway	↑	Induced apoptosis and autophagy in PCa cells -Suppressed tumor cell proliferation.	[225]
Neuregulin	In vitro	LNCaP	-	Activating JNK and Beclin 1	↑	Induced incomplete autophagy and cell death in ROS level-dependent manner - Autophagy activation was independent of mTOR inhibition.	[226]
Rottlerin	In vitro	Human prostate CSCs	-	Activating the AMPK pathway Inhibiting the Akt/mTOR pathway	↑	Induced autophagy, apoptosis, and cytoplasmic vacuolation in prostate CSCs	[229]
Quercetin	In vitro	PC3	-	Inhibiting the PI3K/AKT/mTOR pathway	↑	Inhibited cell viability in a dose-time dependent manner - Induced apoptosis	[230]
Low frequency ultrasound	In vitro	PTX-resistant PC3	-	ERs-mediated downregulation of the PI3K/AKT/mTOR pathway	↑	Induced apoptosis and autophagy- Enhanced chemosensitivity	[231]
Propranolol + 2DG (glycolysis inhibitor)	In vitro/In vivo	PC3, LNCaP, and PNT1A (human immortalized prostatic cell line)	NOD SCID mice bearing PC3 xenografts	-	↓	In vitro: prevented cancer cell proliferation, induced cell apoptosis, altered the morphology of mitochondria, inhibited mitochondrial energetics, and aggravated ERs. In vivo: inhibited tumor growth	[232]
Hydroxytyrosol	In vitro	PC3	-	-	↓	Induced apoptosis and mitochondrial malfunction in PC3 cells through producing superoxide	[233]
BEZ235 or PI103 (Dual PI3K/mTOR inhibitors)	In vitro	PC3 RR, DU145RR and LNCaPRR	-	Inhibiting the PI3K/AKT/mTOR pathway	↓	Improved radiosensitivity via increased rate of apoptosis, inhibition of autophagy, and suppression of the DNA repair mechanisms (NHEJ and HR)	[234]
Cytolethal distending toxin	In vitro	LAPC4	-	Downregulating c-Myc expression Reducing HMGB1	↓	Improved radiosensitivity - Prolonged radiation-induced DSBs – Suppressed autophagy	[235]

↑: increase or induction; ↓: decrease or inhibition; PCa: prostate cancer; 3-MA: 3-methyladenine; CSCs: cancer stem cells; PTX: paclitaxel; ERs: endoplasmic reticulum stress; DSBs: double-strand breaks; NHEJ: non-homologous end joining; HR: homologous recombination; HMGB1: high-mobility group box 1.

## Conclusion and future perspectives

The roles of autophagy in male reproductive biology are increasingly being uncovered, particularly with respect to spermatogenesis and testosterone production - two processes that determine male sexual and reproductive performance. Disorders in the male reproductive system, including PCa, are closely associated with numerous comorbidities that affect the overall quality of life. Therefore, the development of drugs that can regulate autophagy may offer a glimmer of hope for men suffering from these disorders. Although the involvement of autophagy in the physiology and pathophysiology of the male reproductive system, a comprehensive and complete understanding of its contribution to male reproductive system development is first required before novel and effective therapeutic strategies can be planned. However, this area of research is still relatively new and requires further in-depth studies. For example, the exact role of autophagy in many reproductive diseases is not yet fully understood. This is particularly true for PCa, where autophagy is known to have both tumor-promoting and tumor-suppressing functions. It also needs to be clarified whether autophagy activation is a trigger or a consequence of the disease. In addition, current therapies targeting autophagy have focused mainly on phytochemicals, which generally have low bioavailability and target specificity [236]. Many of the autophagy modulators mentioned above are known for their limited selectivity, inconsistent distribution, and rapid excretion [237]. Therefore, future studies should investigate methods that can improve the efficacy of current treatment, such as the delivery of the aforementioned autophagy modulators using nanoparticles. In addition, small molecule drugs and genetic modifications using CRISPR-Cas/siRNAs/shRNAs are considered promising agents for treating various diseases [238, 239], but their utility as autophagy regulators has not been well explored. Therefore, exploring these approaches as treatment alternatives is an interesting avenue for future research. Besides, as autophagy also occurs constitutively in normal physiological functions, it is unknown whether interventions regulating this process would result in toxicity or undesirable side effects. Well-designed in vivo studies and randomized controlled trials are needed to determine the side effects and efficacy of such therapies.

## Data Availability

All data relevant to this review are included in the text, references, table, and figures.
